# Supplementary material for: Tardigrade-Derived Strategy for Low-Cost Storage of Cell-Free Expression Lysates
Source: ACS Synth Biol. 2026 Jun 19;15(7):3061–73. doi: 10.1021/acssynbio.6c00281 (PMC13386639; doi:10.1021/acssynbio.6c00281)
Supplement: Supplementary file 1 [file sb6c00281_si_001.pdf]

Supporting Information

Tardigrade-Derived Strategy for Low-Cost Storage of Cell-Free Expression Lysates

Marten Meckelburg*[1], Imre Banlaki*[1], Aukse Gaizauskaite[1], Henrike Niederholtmeyer[1]**

[1]Technical University of Munich, Campus Straubing for Biotechnology and Sustainability, 94315 Straubing, Germany

*Shared first authorship

**Corresponding author: henrike.niederholtmeyer@tum.de

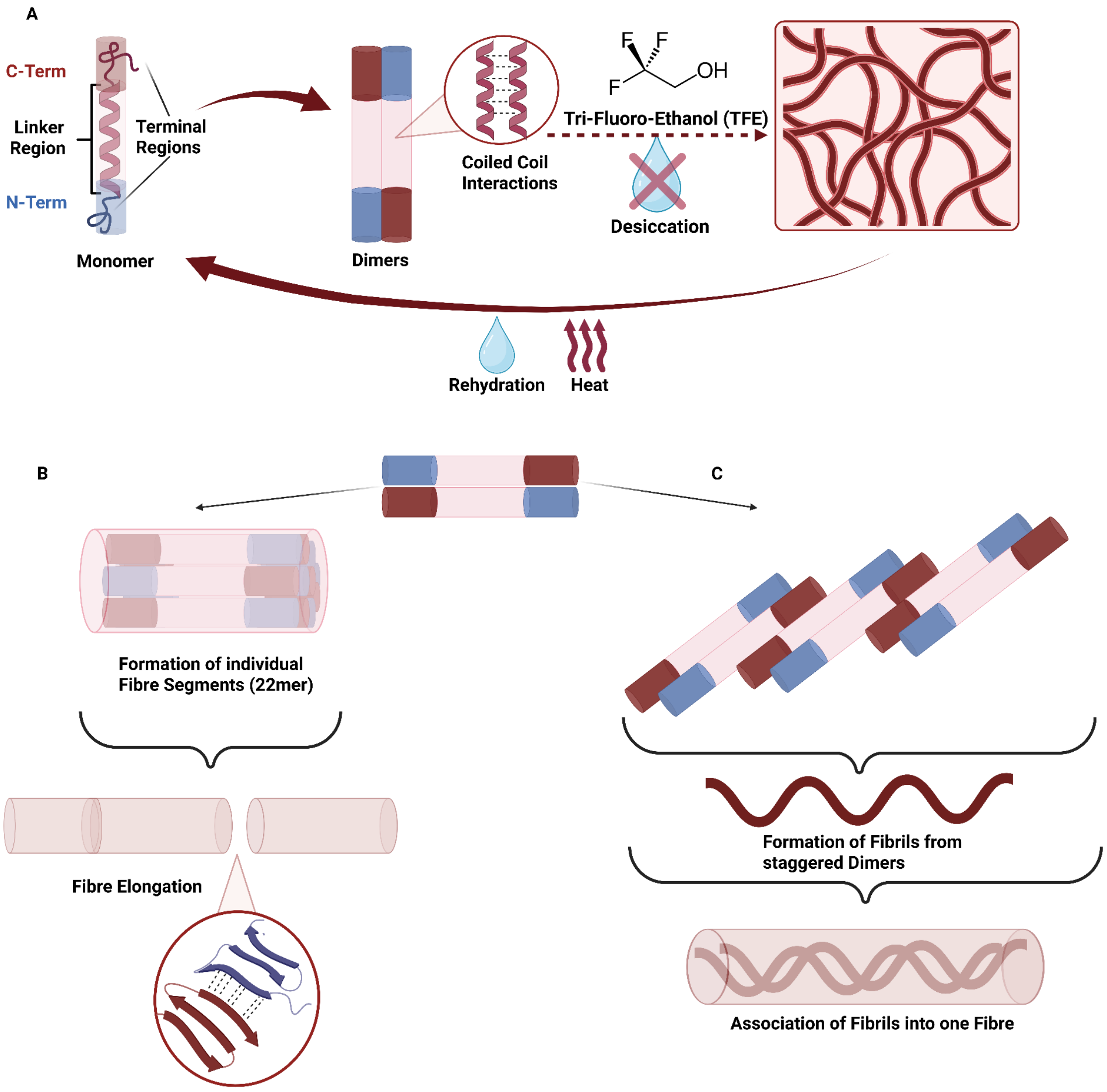

Figure S1. Current models of CAHS protein assembly into higher-order structures during desiccation
(A) Simplified visualisation of a CAHS monomer, highlighting essential structural features. Antiparallel association of monomers into dimers, driven by coiled coil interaction of the linker regions, and further assembly into fibrous networks during desiccation[1]. **(B)** Proposed oligomerisation of dimers into 22-mer fibre bundles and elongation of fibres mediated through ß-ß interactions of "sticky" terminal regions[1]. **(C)** Alternative model, proposing polymerisation of dimers into fibrils which associate to form higher-order fibres[2].

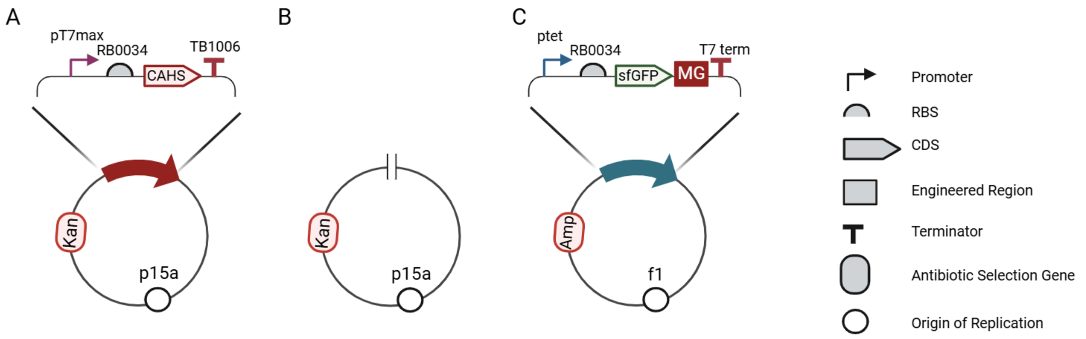

Figure S2. Plasmid maps of constructed vectors

(A) Vector used for the expression of CAHS proteins (used for lysate generation as well as protein purification) **(B)** Empty Vector used for the generation of a control Lysate (EL) lacking the CAHS protein. **(C)** Plasmid used as the DNA template coding for the sfGFP translational reporter and malachite green (MG) aptamer transcriptional reporter used in the assessment of lysate activity. RBS = Ribosome Binding Site, CDS = Coding Sequence, MG = Malachite Green, Kan = Kanamycin, Amp = Ampicillin.

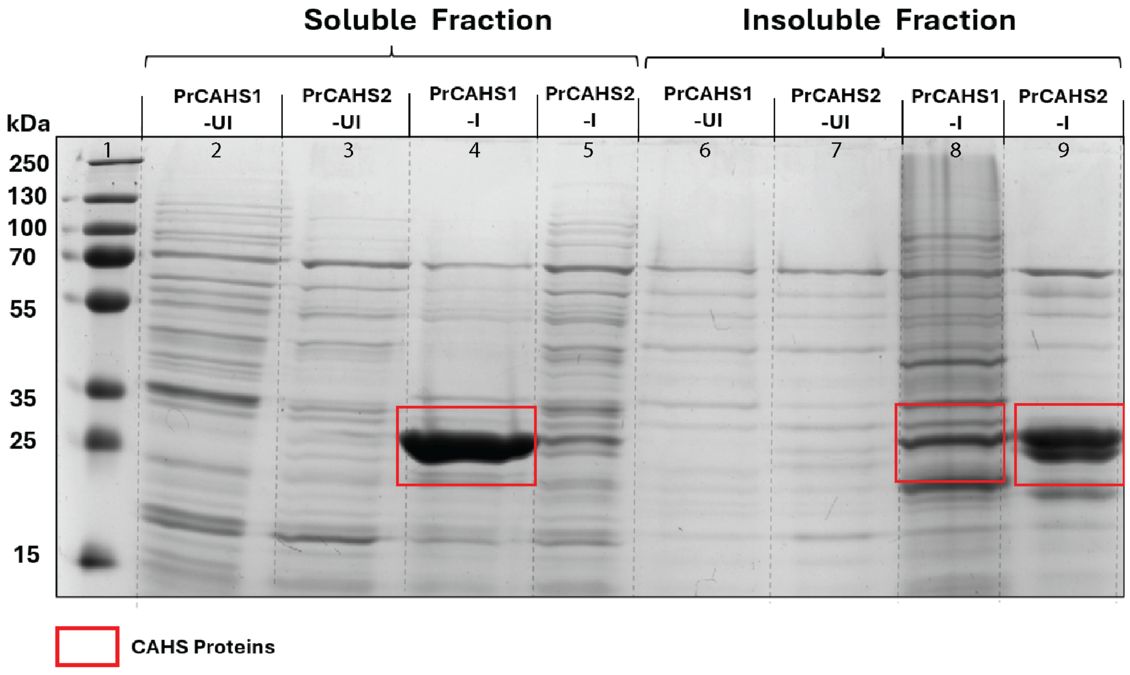

Figure S3. 12% SDS-PAGE assessing heterologous CAHS protein production in *E. coli* BL21(DE3)

Lanes: (1) PageRuler™ prestained protein ladder (Thermofischer Scientific); (2-5) Protein Extracts from soluble fraction (5 µl supernatant following lysis); (6-9) Protein samples (5 µl) generated from insoluble fractions (Pellet following lysis, resolubilized using urea (see Methods)). (2-3,6-7) Protein samples (5 µl) taken immediately prior to induction at OD600=0.2 (4-5,8-9). Samples taken following four hours of induction (1mM IPTG) (5 µl). Abbreviations: PrCAHS1 = Samples taken from *E. coli* BL21(DE3) cultures harboring the PrCAHS1 expression vector, PrCAHS2 = Samples taken from *E. coli* BL21(DE3) cultures harboring the PrCAHS2 expression vector , UI = uninduced, I = Induced Expected size: PrCAHS1 = 26.5 kDa, PrCAHS2 = 25.5 kDa

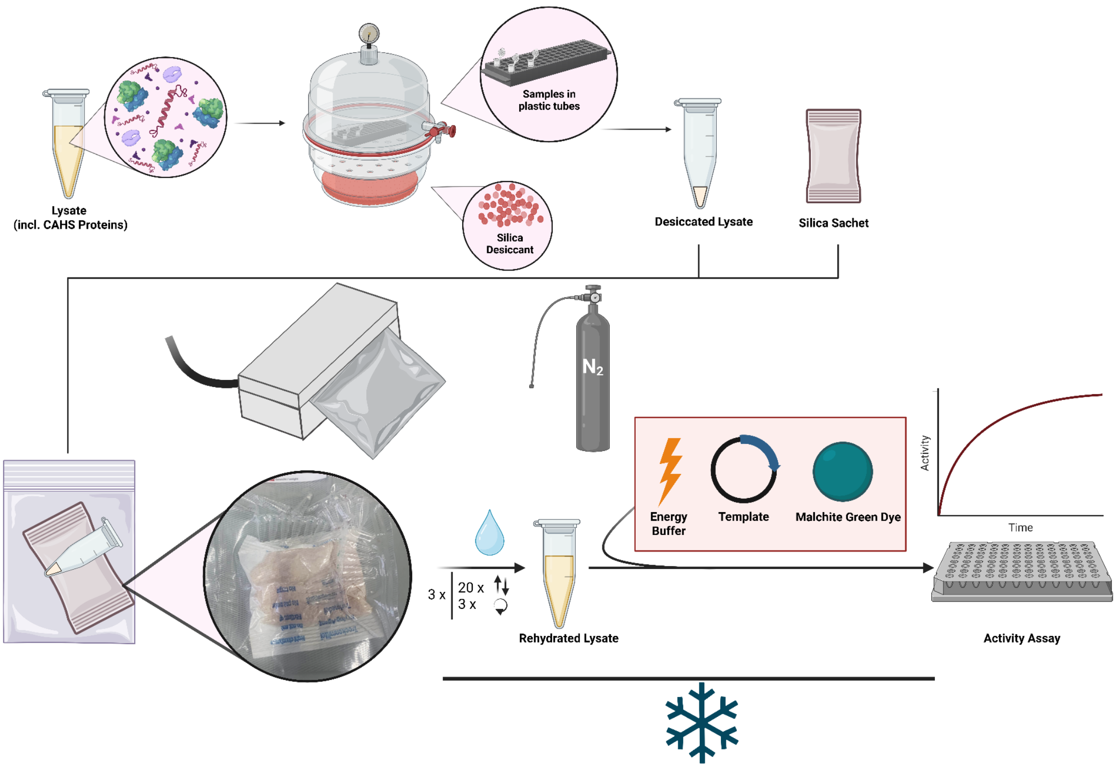

Figure S4. Workflow for the low-cost desiccation of cell-free expression lysates
Open tubes of lysates containing CAHS protein (or controls) were placed inside a conventional vacuum dessicator containing silica dessicant. Following dessication at 11 mbar, at room temperature, the dessicated samples were placed inside a plastic bag with a silica sachet, flushed with liquid nitrogen, and subsequently vacuum sealed utilising an impulse vacuum sealer. After storage at room temperature, samples were rehydrated in a cold room to the original volume using deionised water and mixed thoroughly as indicated. The rehydrated lysate is supplemented with corresponding volumes of energy buffer, plasmid DNA template and malachite green dye, and finally transferred into a plate reader where the activity is measured.

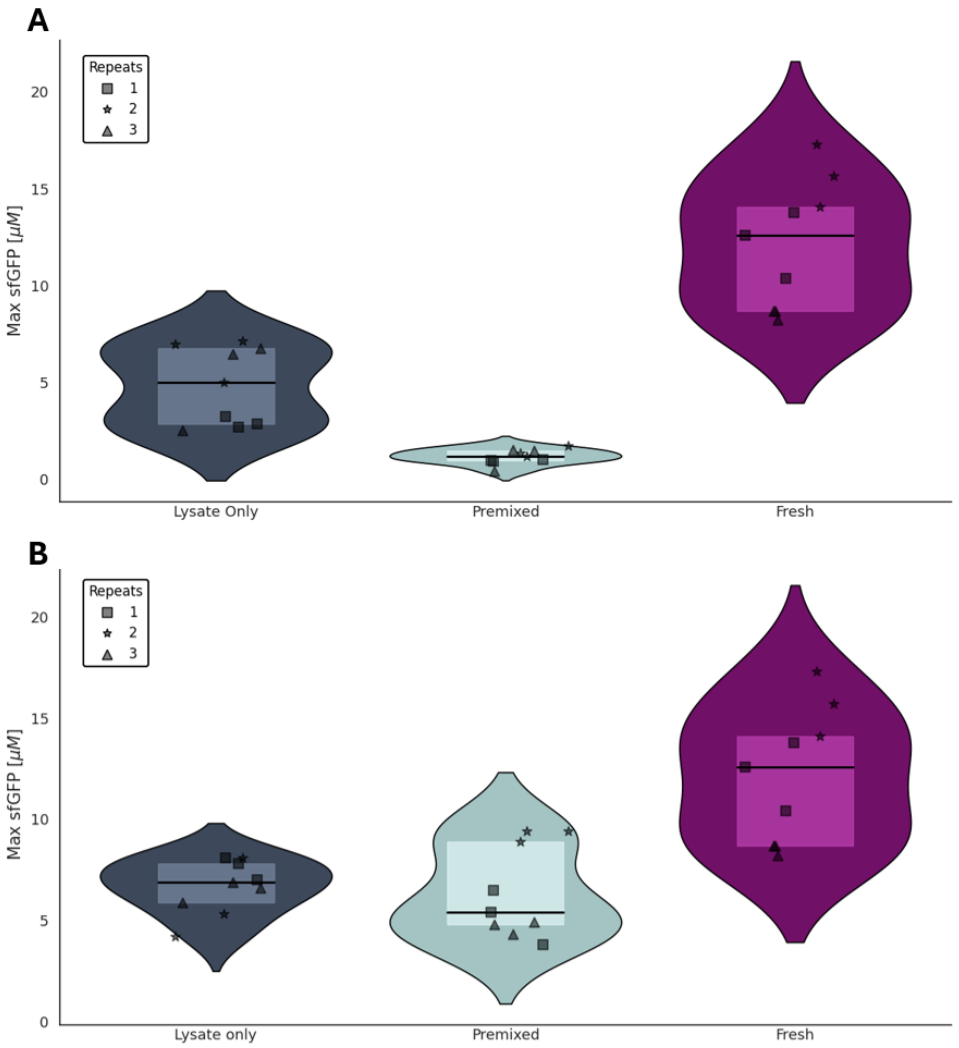

Figure S5. Comparison of different drying methods

Violin plots showing the maximum sfGFP concentrations in vacuum-desiccated **(A)** or lyophilised **(B)** lysates (lystate-only vs. premixed with energy buffer), alongside the corresponding fresh lysates. Symbols indicate individual experiments (n = 9 total across three independent experiments using separate lysate batches as biological replicates with n = 3 technical replicates each). The horizontal line within each violin marks the median, while the upper and lower edges of the box denote the upper and lower quartiles, respectively. “Lysate only” refers to samples in which only the lysate was dried and supplemented with fresh energy buffer just prior to measurement. “Premixed” refers to samples in which the lysate was co-desiccated or co-lyophilised with the energy buffer and rehydrated prior to measurement.

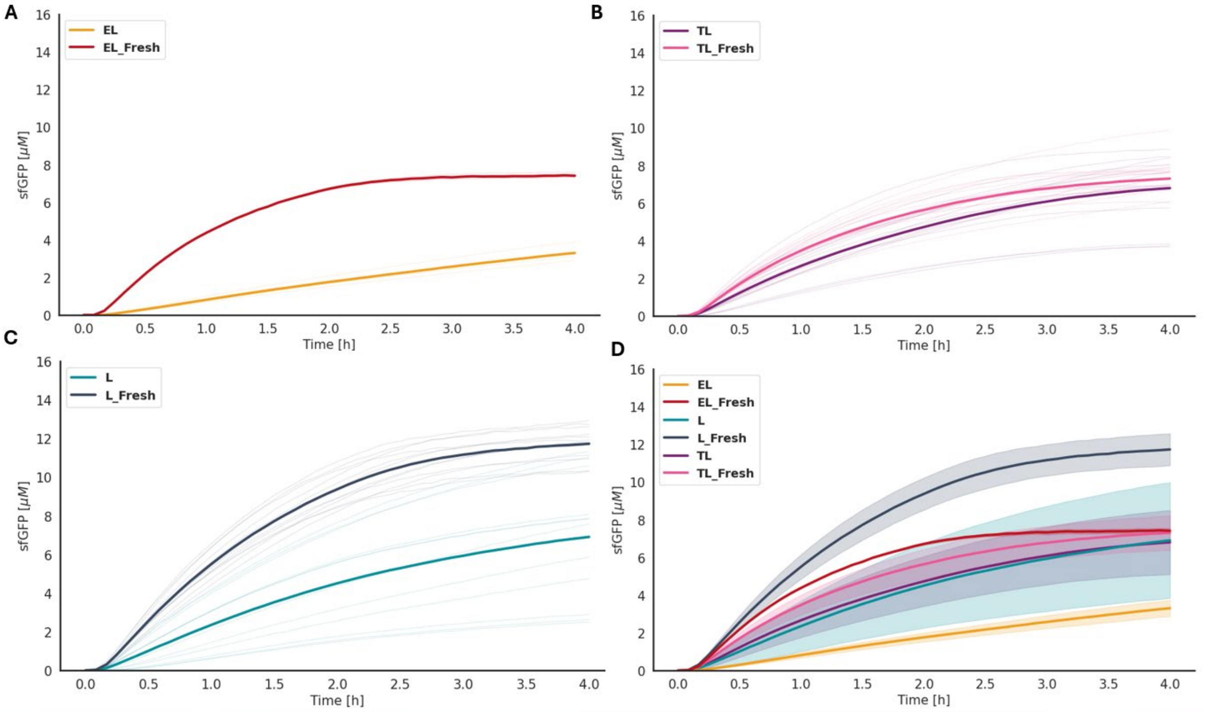

Figure S6. Protein synthesis kinetics for different lysate types, comparing desiccated and fresh samples individually

(A-C) sfGFP synthesis kinetics for different lysate types (control lysate EL **(A)**, tardigrade lysate TL **(B)** and standard lysate L **(C)**) and treatments (desiccated and fresh). Solid thick lines show mean values and thinner lines show individual replicates from four short-term desiccation experiments (n = 12, across four experiments with n = 3 technical replicates, respectively or n = 3 technical replicates for **A**). Data from Figure 2A is shown individually for different lysate types. **(D)** sfGFP synthesis kinetics for the data shown in **(A-C)** combined, showing the standard deviation of individual replicates as error bands. Tardigrade lysate (TL), produced from a strain expressing PrCAHS1 protein; Empty vector control lysate (EL), produced from a strain carrying the autolysis plasmid plus the empty expression vector.

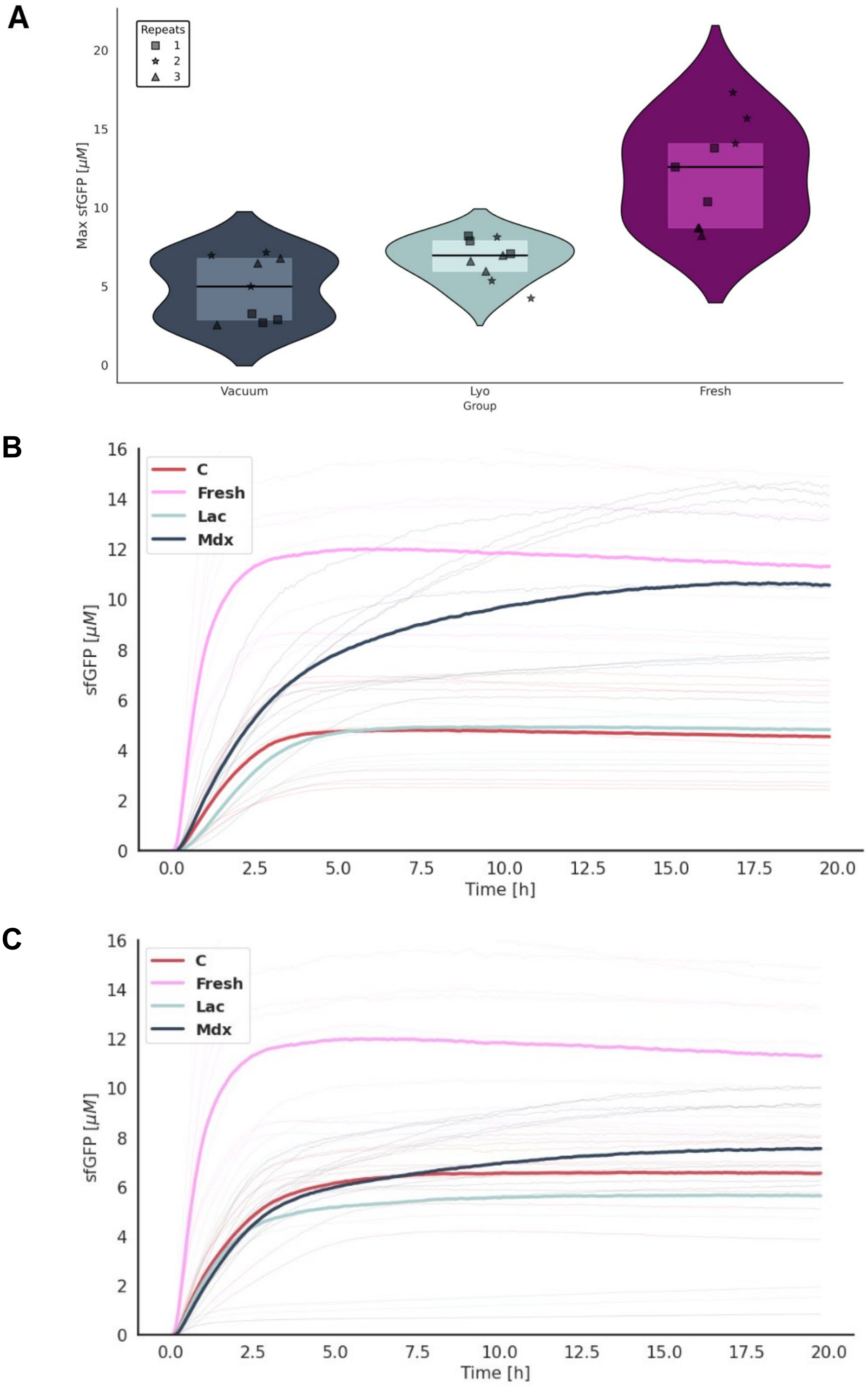

Figure S7. Desiccation compared to lyophilisation in the presence of sugar additives
(A) Violin plots showing the maximum sfGFP concentrations in vacuum-desiccated (Vacuum) or lyophilised (Lyo) lysate samples, alongside the corresponding fresh lysates. Symbols indicate individual experiments (n = 9 total, across three independent experiments using separate lysate batches as biological replicates, with n = 3 technical replicates each). The horizontal line within each violin marks the median, while the upper and lower edges of the box denote the upper and lower quartiles, respectively. **(B-C)** sfGFP synthesis kinetics for samples subjected to drying with different additives (Lactose (Lac), Maltodextrin (Mdx) or none (Control, C). We compared two drying methods, vacuum desiccation **(B)** and lyophilisation **(C)**. Solid thick lines show mean values and thinner lines show individual replicates from four 1-week desiccation experiments (n = 9 total, across three independent experiments using separate lysate batches as biological replicates, with n = 3 technical replicates each).

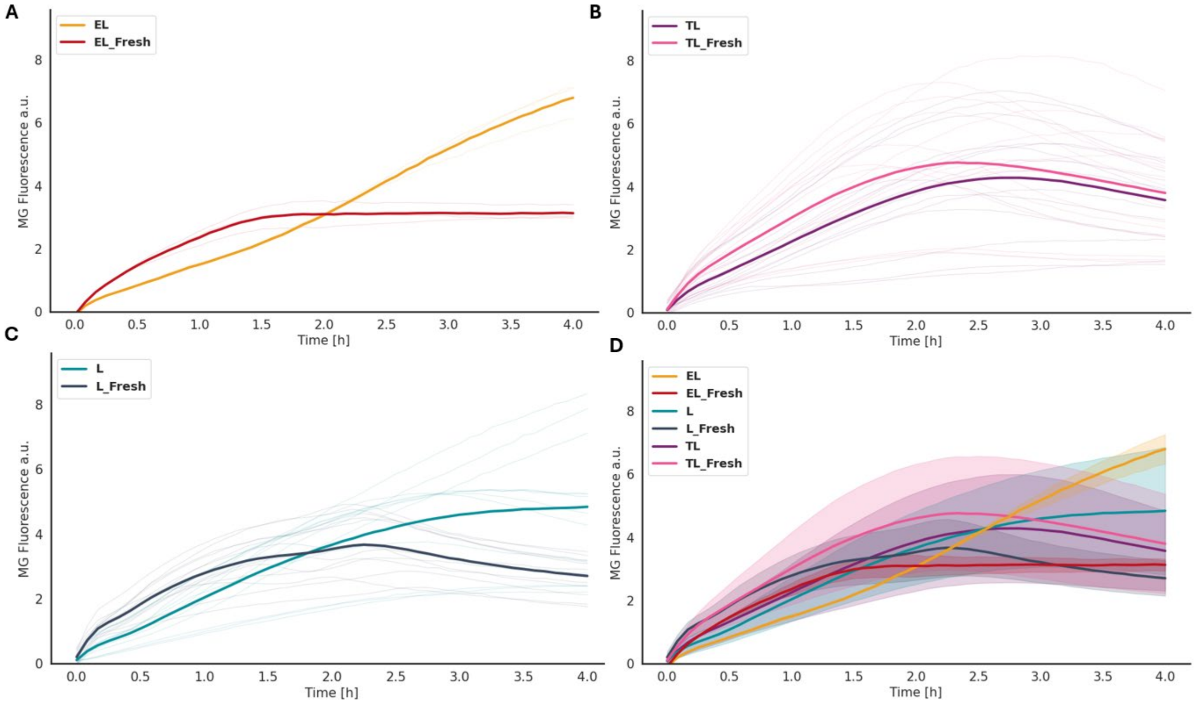

Figure S8. MG kinetics for different lysate types, comparing desiccated and fresh samples individually
(A-C) Malachite green (MG) fluorescence kinetics for different lysate types (control lysate EL **(A)**, tardigrade lysate TL **(B)** and standard lysate L **(C)**), and treatments (desiccated and fresh). Shown is MG fluorescence data of four separate 1-week desiccation experiments. Solid thick lines show mean values, and thin lines correspond to individual replicates from four short-term desiccation experiments (n = 12 per group, except EL (n = 3)). Data from Figure 3A is shown individually for different lysate types. **(D)** MG fluorescence kinetics for the data shown in **(A-C)**, showing the standard deviation of individual replicates as error bands. Tardigrade lysate (TL), produced from a strain expressing PrCAHS1 protein; Empty vector control lysate (EL), produced from a strain carrying the autolysis plasmid plus the empty expression vector.

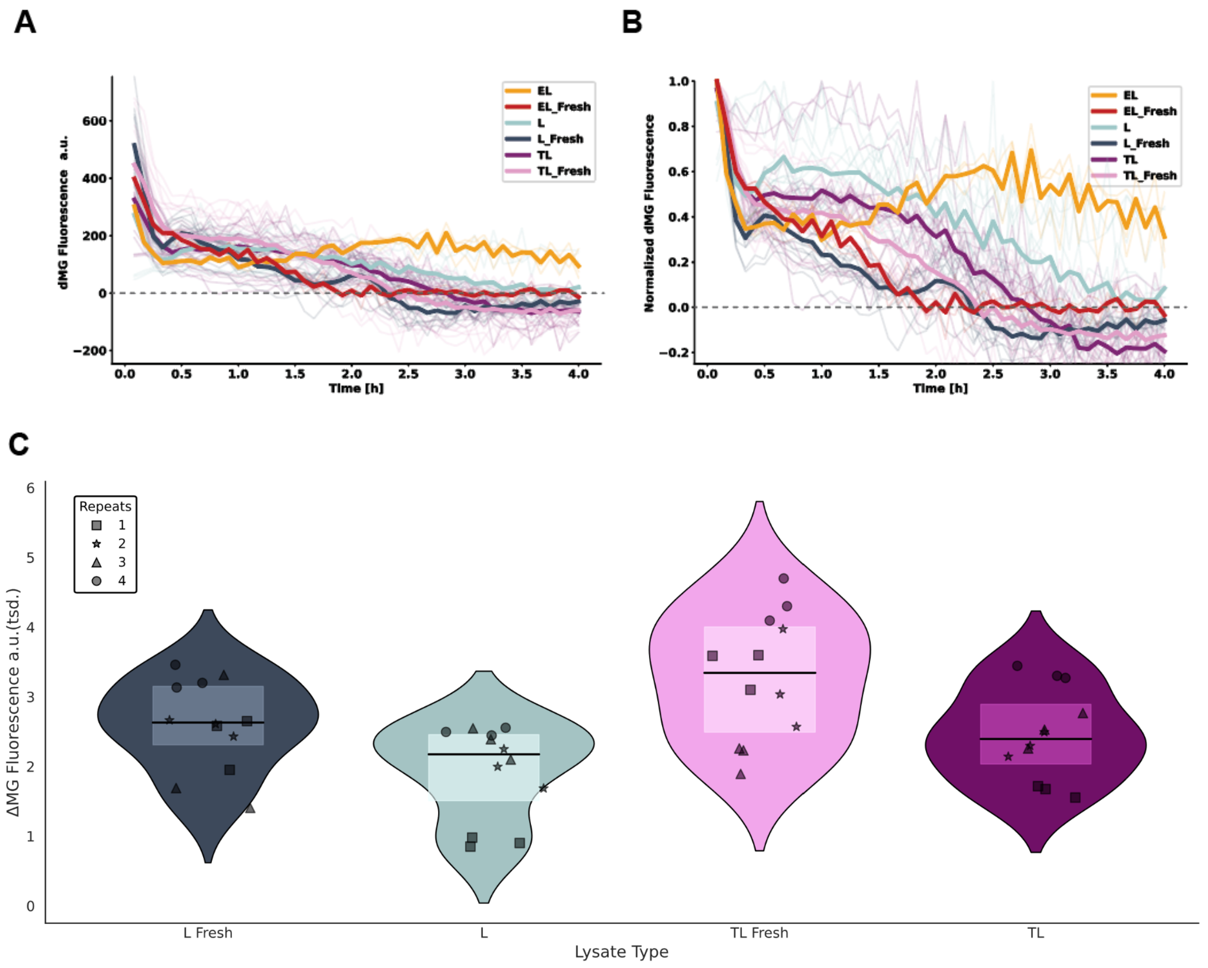

Figure S9. Rate of change in MG signal kinetics

(A) ΔMG kinetics of different lysate types and treatment. Bold lines are the mean values of replicates. The dashed, grey line marks the inversion where the MG signal started to decrease over time. **(B)** Normalised ΔMG kinetics emphasise the different trends over time between fresh, intact and degraded lysates. Running ΔMG kinetics were calculated by subtracting MG fluorescence at time point n (n times 5 minutes) from the preceding value at time point n-1. **(C)** Initial mRNA synthesis rates. Violin plot comparing ΔMG between 1h and 0h across four separate 1-week desiccation experiments. Symbols distinguish individual experiments (n = 12). The line within the violin plot represents the median, upper and lower borders of the box plots representing upper and lower quartiles, respectively.

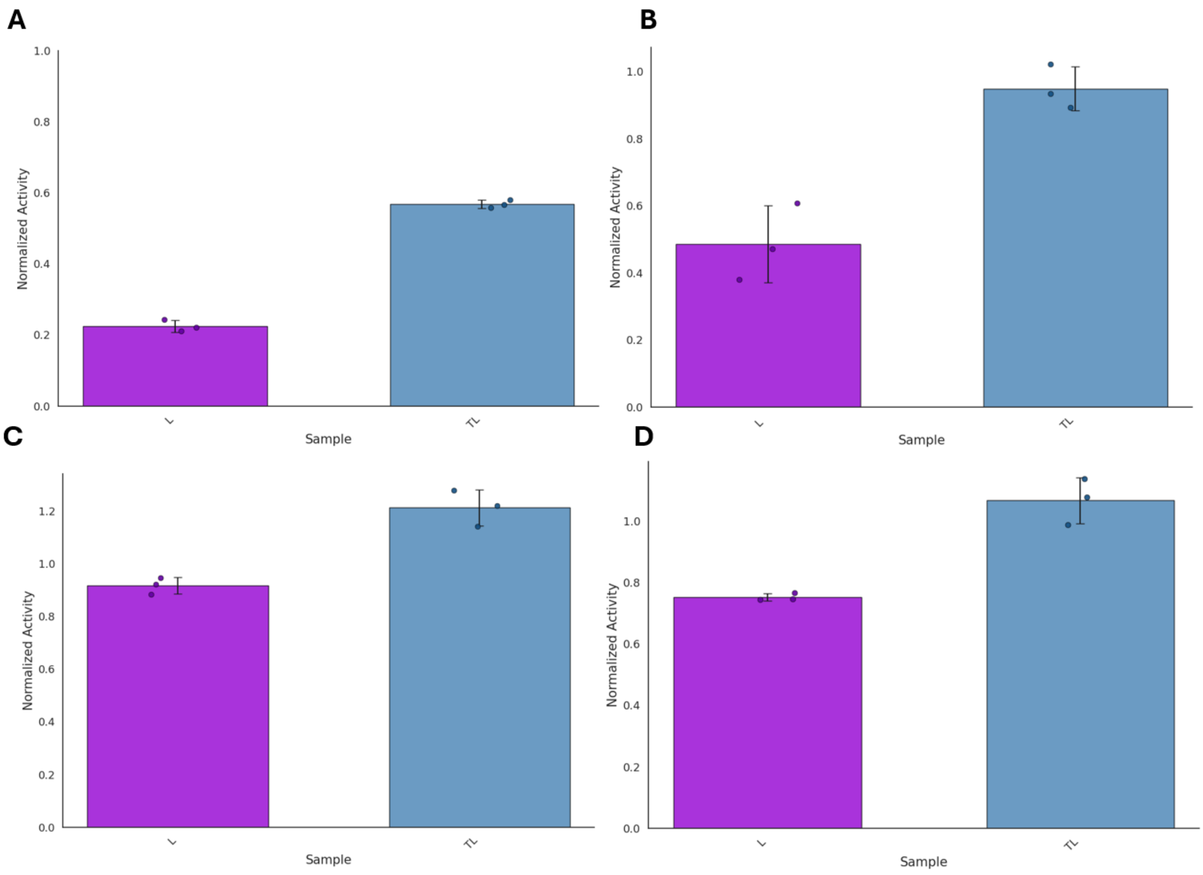

Figure S10. Normalised maximum sfGFP production in individual short-term desiccation experiments (A-D) Maximum normalised sfGFP concentrations of different lysate types L (purple) and TL (blue) after 1 week of desiccation. Bars show means ± SD (n = 3). Individual data points are overlaid. **(A-D)** are the results from separate desiccation experiments in Fig. 2B (01-04).

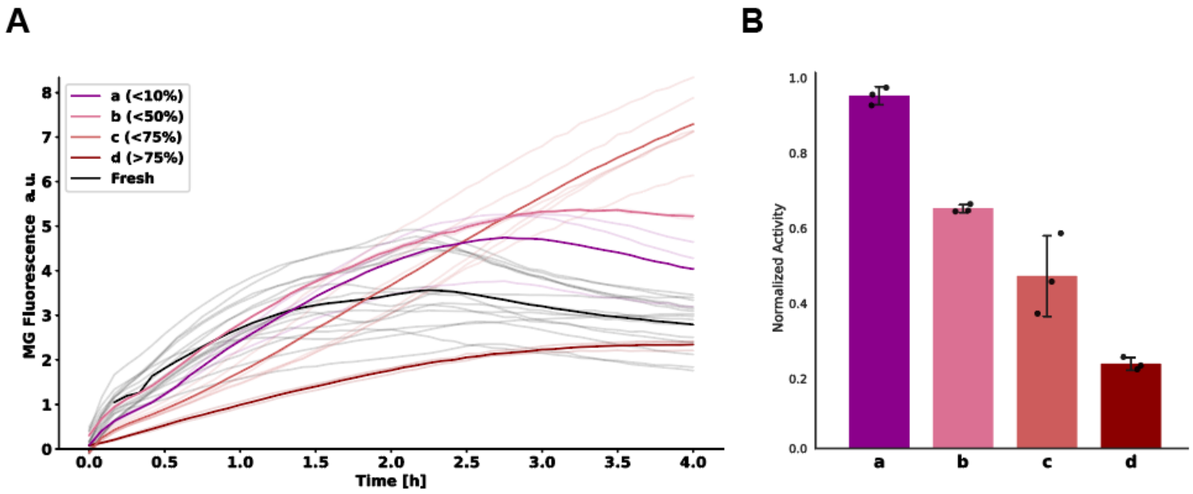

Figure S11. MG kinetics from individual short-term desiccation experiments grouped by damage
(A) Malachite Green (MG) fluorescence kinetics across four separate short-term (1 week) desiccation experiments of standard lysate not containing tardigrade CAHS proteins. Solid lines represent means, faint traces represent individual replicates. Colours and letters denote damage categories defined by the inverse of normalised sfGFP concentration from the corresponding samples. (a) <10% damage, (b) 10–50%, (c) 50–75%, and (d) >75%. **(B)** Normalised activity of rehydrated standard lysates calculated from endpoint sfGFP signal.

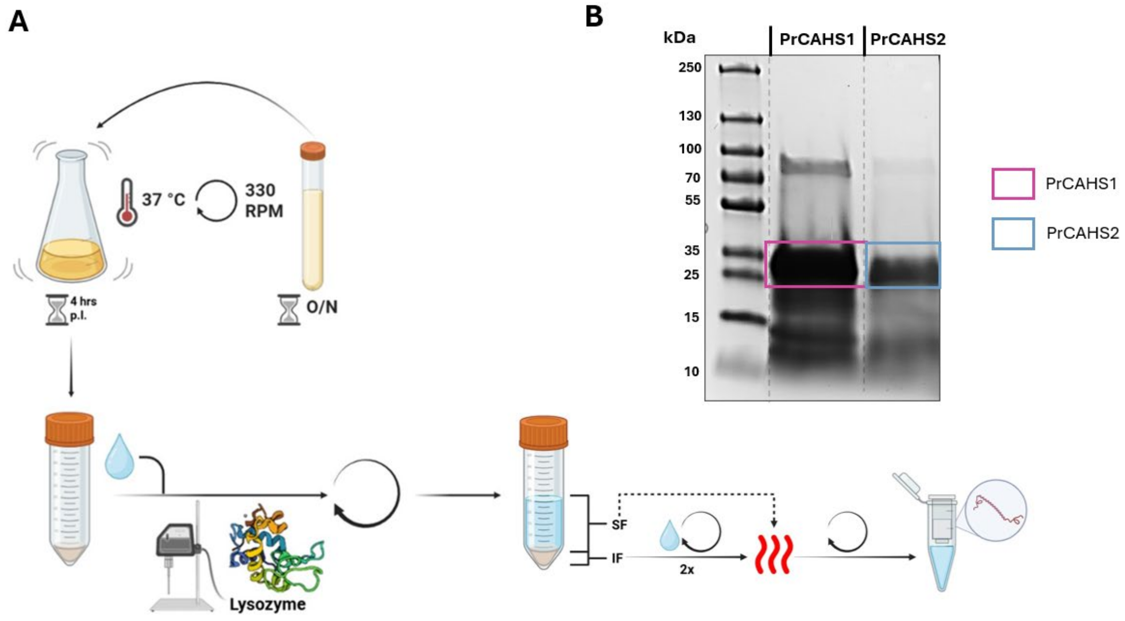

Figure S12. Workflow for the purification of CAHS proteins
(A) Schematic of the CAHS protein purification workflow. Overnight cultures (5 mL) of *E. coli* BL21(DE3) cells carrying the CAHS expression construct (table S1) were used to inoculate 250 mL LB cultures. Following induction at OD_{600} = 0.3–0.4, cells were incubated for 4 h before biomass collection. Pelleted cells were resuspended in buffer (composition variable) and lysed by combined lysozyme treatment and ultrasonication. Soluble and insoluble fractions were separated by centrifugation. After multiple washes of the insoluble fraction, both soluble and insoluble fractions were subjected to heat treatment (95 °C, ≤90 min). CAHS proteins remained in the soluble fraction after heat treatment and were further concentrated using Amicon® filter units. **(B)** Exemplary SDS page (12 %) showing the CAHS proteins extracted from *E. coli* BL21(DE3) using the established purification protocol. (5 µl of samples were loaded); Lanes: (1) PageRuler™ prestained protein ladder (Thermofischer Scientific); (2) PrCAHS1 (5 µl); (3) PrCAHS2 (5 µl). Expected size: PrCAHS1 = 26.5 kDa, PrCAHS2 = 25.5 kDa

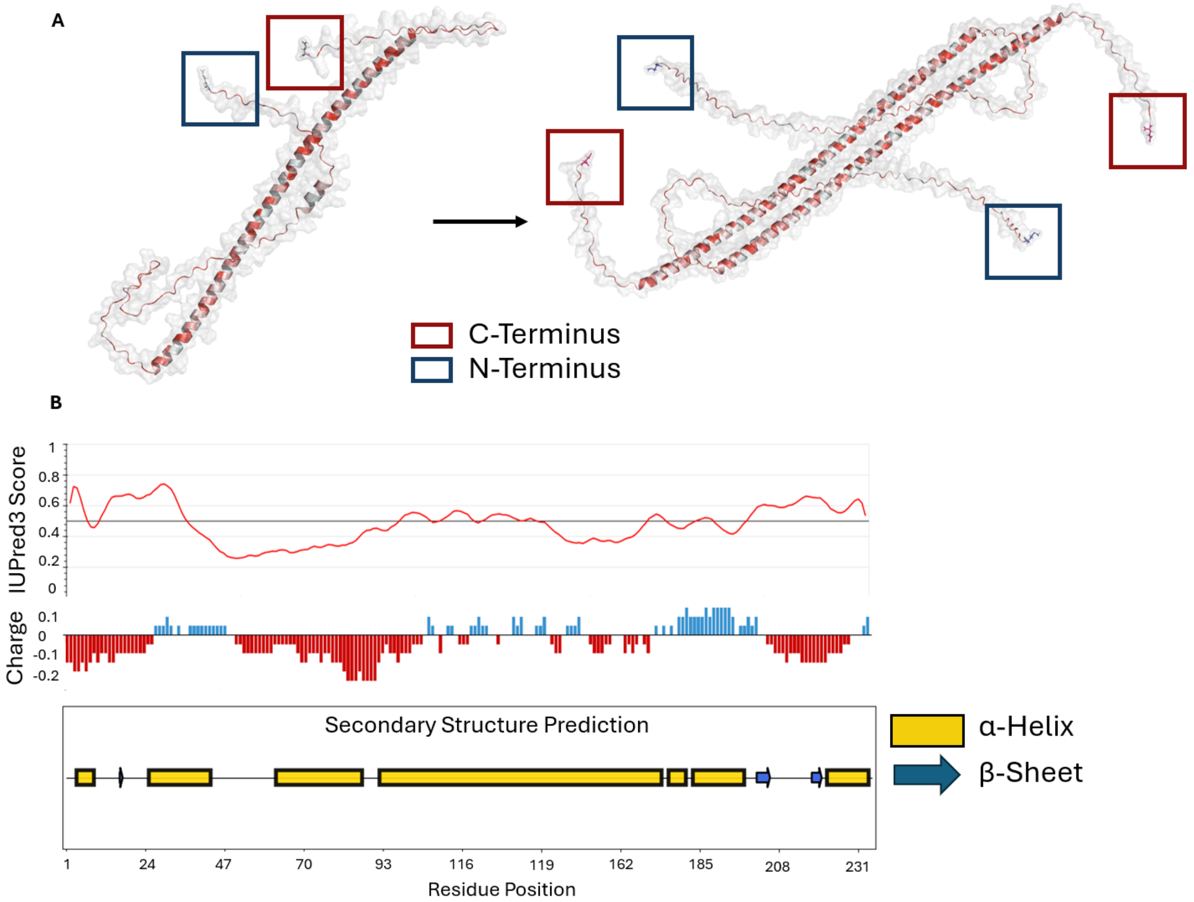

Figure S13. *In silico* modeling of PrCAHS1
(A) PyMOL visualisation of AlphaFold2 predictions of PrCAHS1 secondary structure (monomeric) and in a dimeric state. Termini are indicated with blue (N-Terminus) and red (C-terminus), respectively. Individual residues were colored in a hydrophobic gradient: non-hydrophobic (white) to hydrophobic (red) amino acids[3]. **(B)** Sequence property predictions. Top: intrinsic disorder of residues predicted using IUPRED 3 webserver[4]. Middle: Charge of individual residues estimated using the Protein-Sol software[5]. Bottom: Secondary Structure prediction of PrCAHS1 using the PSSpred functionality of the MPI bioinformatics toolkit platform[6,7].

Table S1. Plasmids used in this study: promoter, RBS, gene, terminator, gene sequences are capitalised

Plasmid	Description	Reference	Relevant sequence
**pCAHS1**<br>(Addgene #255769)	Plasmid used for the heterologous expression of PrCAHS1 for purification and lysate generation.<br><br>Components:<br>pJUMP26-1A(sfGFP)_p**T7max**::**RB0034**::**PrCAHS1**::**TB1006**, Kanamycin Resistance, p15a	this study	**gcctttcgttttatttgatgcctttaattaaggagttttgcaggtgccttggaac<br>acctgcttttcgctgaattcgcggccgcttctagagcgtctctggagAATT<br>CTAATACGACTCACTATAGGGATACTAGAGAAAGAGG<br>AGAAATAATCAATGTCAGCGGAAGCTATGAACATGAA<br>CATGAACCAGGATGCAGTTTTTATTCCTCCTCCGGAA<br>GGCGAACAGTATGAACGCAAAGAGAAACAAGAGATT<br>CAACAGACGTCGTACCTGCAAAGCCAGGTAAAGGTT<br>CCGCTGGTCAACCTGCCCGCACCTTTCTTCTCAACCT<br>CCTTCTCCGCGCAGGAGATCCTTGGGGAAGGTTTCCA<br>GGCTAGTATTTCTCGGATTTCCGCCGTTAGTGAAGAA<br>CTGTCCTCTATCGAAATTCCGGAGCTGGCGGAAGAGG<br>CCCGTCGTGACTTTGCGGCCAAAACGCGCGAACAGG<br>AGATGCTGTCTGCTAACTATCAAAAAGAAGTTGAGCG<br>CAAAACTGAAGCCTATCGGAAGCAGCAAGAAGTGGA<br>GGCAGACAAAATTCGGAAAGAGCTGGAAAAGCAGCA<br>TCTGCGTGACGTGGAGTTCCGCAAAGATATTGTAGAG<br>ATGGCAATCGAAAACCAGAAAAAAATGATTGATGTCG<br>AAAGCCGTTATGCAAAAAAAGATATGGACCGCGAAC<br>GTGTGAAAGTCCGTATGATGCTCGAGCAGCAAAAATT<br>TCATAGCGACATCCAGGTAAATCTCGATTCTAGCGCA<br>GCAGGGACCGAAACTGGAGGCCAAGTTGTTTCAGAA<br>TCTCAGAAATTTACCGAACGTAATCGTCAGATTAAAC<br>AGGCTTAAAAAAAAAAACCCCGCCCCTGACAGGGCG<br>GGGTTTTTTTTcgctaatgtgagacgtactagtagcggccgctgcag<br>ggagttgtcttcgaagacttcgctctagtcttggactc**
pCAHS2	Plasmid used for the heterologous expression of PrCAHS2 for purification.<br><br>Components:<br>pJUMP26-1A(sfGFP)_p**T7max**::**RB0034**::**PrCAHS2**::**TB1006**, Kanamycin Resistance, p15a	this study	**gcctttcgttttatttgatgcctttaattaaggagttttgcaggtgccttggaac<br>acctgcttttcgctgaattcgcggccgcttctagagcgtctctggagAATT<br>CTAATACGACTCACTATAGGGATACTAGAGAAAGAGG<br>AGAAATAATCAATGGAGGCCATGAATATGAATATCCC<br>CCGCGATGCCATGTTTGTTCCGCCACCGGAATCTGAG<br>CAAAATGGGTATCATGAGAAGTCAGAAGTTCAGCAAA<br>CAAGTTATATGCAGAGTCAAGTCAAAGTGCCACATTA<br>TAATTTCCCGACACCATATTTTACGACTTCCTTTTCTG<br>CGCAAGAGCTGCTGGGCGAAGGGTTTCAAGCCTCAA<br>TTTCCCGTATTTCAGCCGTTACGGAAGACATGCAGAG<br>CATGGAAATCCCGGAGTTCGTTGAAGAGGCCCGCCG<br>TGATTACGCAGCCAAAACACGTGAAAATGAGATGCTG<br>GGGCAACAATATGAAAAAGAGCTGGAACGTAAGTCC<br>GAAGCCTACCGCAAACATCAGGAAGTAGAGGCCGAC<br>AAAATCCGCAAAGAACTTGAAAAACAGCATATGCGTG<br>ATATTGAATTTCGGAAAGAAATTGCAGAACTGGCGAT<br>TGAGAACCAAAAACGTATGATCGATCTTGAATGCCGC<br>TATGCAAAAAAAGACATGGACCGGGAACGCACAAAA<br>GTTCGTATGATGCTGGAGCAACAGAAATTCCATAGTG<br>ATATCCAGGTAAATCTGGATTCTTCTGCGGCTGGGAC<br>CGAGAGCGGAGGTCATGTAGTGAGCCAGTCTGAAAA<br>GTTCACCGAACGTAACCGCGAGATGAAACGCGCTTA<br>AAAAAAAAAACCCCGCCCCTGACAGGGCGGGGTTTT<br>TTTTcgctaatgtgagacgtactagtagcggccgctgcagggagttgtct<br>tcgaagacttcgctctagtcttggactcctgttgatag**
pEmpty	Plasmid used for the generation of the empty vector control lysate.<br><br>Components:<br>pJUMP26-1A(sfGFP), Kanamycin Resistance, p15a	this study	

**psfGFP-MG** (Addgene #255770)	Dual reporter plasmid to evaluate effects of desiccation on transcription and translation<br><br>Components:<br>MC_2-5-DO**_pTet::RBS**::**sfGFP**::3xSTOP::**MG**:**:T7terminator**<br>Ampicillin Resistance, ColE1	this study	**ggagaggcggttgtagtggggggaatggatagcaagctgcgggtagac<br>ctccaattattgaaggcctcccaaatcggggggccttttttattgataacaaa<br>aGGAGCTGTTCTCAGTGATAGAGATTGACATCCCTAT<br>CAGTGATAGATATAATGAGCACTACTAGAGAGAAGGA<br>GGAAAAAAAAATGCGTAAAGGTGAAGAACTGTTTACC<br>GGTGTTGTTCCAATTCTGGTTGAACTGGATGGTGATGT<br>TAACGGTCACAAATTTTCTGTTCGTGGTGAAGGCGAA<br>GGTGATGCAACCAACGGTAAACTGACCCTGAAATTTA<br>TCTGTACCACTGGTAAACTGCCAGTTCCATGGCCAAC<br>TCTGGTTACCACTCTGACCTACGGTGTTCAATGTTTTG<br>CACGTTACCCAGATCACATGAAACAACACGATTTTTT<br>CAAAAGCGCAATGCCAGAAGGTTACGTTCAAGAACG<br>TACCATCTCTTTTAAAGATGACGGCACCTACAAAACC<br>CGTGCGGAAGTTAAATTTGAAGGTGATACCCTGGTTA<br>ACCGCATTGAACTGAAAGGCATCGATTTTAAAGAAGA<br>TGGTAACATCCTGGGCCACAAACTGGAATACAACTTT<br>AACTCTCACAACGTGTACATCACCGCAGACAAACAAA<br>AAAACGGTATCAAAGCGAACTTCAAGATCCGTCACAA<br>CGTTGAAGATGGTTCTGTTCAACTGGCAGATCACTAC<br>CAACAAAACACCCCAATTGGTGATGGTCCAGTTCTGC<br>TGCCAGATAACCACTACCTGTCTACCCAAAGCGTTCT<br>GTCTAAAGATCCAAACGAAAAACGTGATCACATGGTG<br>CTGCTGGAATTTGTTACCGCAGCAGGTATTACCCACG<br>GTATGGATGAACTGTACAAAGCAGCTTTATGATGATG<br>AGGGTATGCCTGGCGACCATAGCGATTGGGTAACCG<br>GATCCCGACTGGCGAGAGCCAGGTAACGAATGGATC<br>CGGTAACCAATTAGCGCCGATGGTAGTGTGGGGTTTC<br>CCCATGTGAGAGTAGGACATCGCCAGGCATTAGCATA<br>ACCCCTTGGGGCCTCTAAACGGGTCTTGAGGGGTTTT<br>TTGcgctggacccgcgtgtcttcggagaaccatctcgtaagaggatagt<br>agttactggagacga**
pAD-LyseR	Used for facile autolysis during lysate generation<br><br>Components:<br>pPro24_beta-lactamase<br>phage lambda gene R	[8]	See Addgene [# 99244]

References

(1) Sanchez-Martinez, S.; Nguyen, K.; Biswas, S.; Nicholson, V.; Romanyuk, A. V.; Ramirez, J.; Kc, S.; Akter, A.; Childs, C.; Meese, E. K.; Usher, E. T.; Ginell, G.; Yu, F.; Gollub, E.; Malferrari, M.; Francia, F.; Venturoli, G.; Martin, E. W.; Caporaletti, F.; Giubertoni, G.; Woutersen, S.; Sukenik, S.; Woolfson, D. N.; Holehouse, A.; Boothby, T.; VI.Veni; Cortajarena, A. Labile Assembly of a Tardigrade Protein Induces Biostasis. *Protein Sci.* **2024**. https://doi.org/10.1002/pro.4941.

(2) Malki, A.; Teulon, J.; Mikkola, E. A.; Maurin, D.; Pellequer, J.; Nanao, M. H.; Blackledge, M. Fibril Structure of Desiccation-Protective Tardigrade Protein CAHS-8. *Angew. Chem. Int. Ed.* **2026**, *65* (7), e19912. https://doi.org/10.1002/anie.202519912.

(3) Hagemans, D.; Van Belzen, I. A. E. M.; Morán Luengo, T.; Rüdiger, S. G. D. A Script to Highlight Hydrophobicity and Charge on Protein Surfaces. *Front. Mol. Biosci.* **2015**, *2*. https://doi.org/10.3389/fmolb.2015.00056.

(4) Erdős, G.; Pajkos, M.; Dosztányi, Z. IUPred3: Prediction of Protein Disorder Enhanced with Unambiguous Experimental Annotation and Visualization of Evolutionary Conservation. *Nucleic Acids Res.* **2021**, *49* (W1), W297–W303. https://doi.org/10.1093/nar/gkab408.

(5) Hebditch, M.; Carballo-Amador, M. A.; Charonis, S.; Curtis, R.; Warwicker, J. Protein–Sol: A Web Tool for Predicting Protein Solubility from Sequence. *Bioinformatics* **2017**, *33* (19), 3098–3100. https://doi.org/10.1093/bioinformatics/btx345.

(6) Yan, R.; Xu, D.; Yang, J.; Walker, S.; Zhang, Y. A Comparative Assessment and Analysis of 20 Representative Sequence Alignment Methods for Protein Structure Prediction. *Sci. Rep.* **2013**, *3* (1), 2619. https://doi.org/10.1038/srep02619.

(7) Zimmermann, L.; Stephens, A.; Nam, S.-Z.; Rau, D.; Kübler, J.; Lozajic, M.; Gabler, F.; Söding, J.; Lupas, A. N.; Alva, V. A Completely Reimplemented MPI Bioinformatics Toolkit with a New HHpred Server at Its Core. *J. Mol. Biol.* **2018**, *430* (15), 2237–2243. https://doi.org/10.1016/j.jmb.2017.12.007.

(8) Didovyk, A.; Tonooka, T.; Tsimring, L.; Hasty, J. Rapid and Scalable Preparation of Bacterial Lysates for Cell-Free Gene Expression. *ACS Synth. Biol.* **2017**, *6* (12), 2198–2208. https://doi.org/10.1021/acssynbio.7b00253.
